# Maximizing tuberculosis services through private provider engagement – A case study from Pakistan

**DOI:** 10.1016/j.jctube.2024.100506

**Published:** 2025-01-20

**Authors:** Aamna Rashid, Surbhi Sheokand, Razia Fatima, Hammad Habib, Adeel Tahir, Asim Saleem, Poshan Thapa, Petra Heitkamp

**Affiliations:** a Private Sector Engagement Consultant – TB, Dar-es-Salaam, United Republic of Tanzania; b Research Institute of the McGill University, Health Centre Montreal QC Canada; c TB PPM Learning Network, McGill International TB Centre, McGill University Montreal QC Canada; d Common Management Unit for AIDS, TB and Malaria, Ministry of National Health Services, Regulations & Coordination, Islamabad, Pakistan; e National TB Control Program, Islamabad, Pakistan; f Mercy Corps, Private Sector Principal Recipient for The Global Fund TB Program, Islamabad, Pakistan; g School of Population and Global Health, McGill University Montreal QC Canada

## Abstract

Pakistan is the fourth highest contributor to the globally estimated 3.7 million tuberculosis (TB) cases. Due to the subpar condition of public sector facilities in Pakistan, the private sector remains the preferred choice, with over 90% of people accessing it for TB care. Aligning with the World Health Organization’s (WHO) patient-centered approach, the private provider engagement program led by Mercy Corps (MC) and supported by the Global Fund has been actively engaging the private sector for over a decade in strengthening Pakistan’s TB services through innovative interventions. Their public–private mix (PPM) strategies like, involving General Practitioners (GPs), large private hospitals, pharmacies, specimen transportation and mobile outreach chest camps, take an integrated approach (Fig. 1) to ensure treatment adherence, completion, and contact screening in reaching the last mile. In this paper, we present MC’s contributions as a case study to elaborate on the crucial role of private provider engagement in improving overall TB care, increasing TB notifications, and addressing the urgent need to identify people with undiagnosed TB.

## 1 Background

Pakistan, the fifth most populous country in the world, has a population density of approximately 300 people per square kilometer. It ranks fifth among the thirty highest TB burden countries globally [1], [2]. As per the World Health Organization (WHO) Global TB Report 2023, the incidence rate of TB in Pakistan is 258 per 100,000 population, meaning one person develops TB every fifty-two seconds [3], representing the significant TB burden in the country. The healthcare system in Pakistan is composed of both public and private sectors, where the primary responsibility of TB-related services lies largely with the government health system [4].

The National TB Control Program (NTP), operating under the Ministry of National Health Services Regulation and Coordination, works in collaboration with the Provincial TB Control Programs to provide free diagnosis and treatment through an extensive network of over 1,700 public and private health facilities. In 2020, the NTP expanded to include a TB Information helpline and a TB Training portal for healthcare providers [5]. In line with the WHO End TB Strategy which emphasizes the inclusion of the private sector within the framework of Public-Private Mix (PPM) in TB care, Pakistan has recently updated its National and Provincial Strategic Plans (2024––2026). These plans aim to guide a diverse range of stakeholders involved in TB care, including government entities, development partners, civil society, international agencies, research institutions, and the private sector. The collaborative effort of these stakeholders has positioned NTP as one of the best-performing public health programs in Pakistan in terms of coverage and impact [6].

**Fig. 1:**
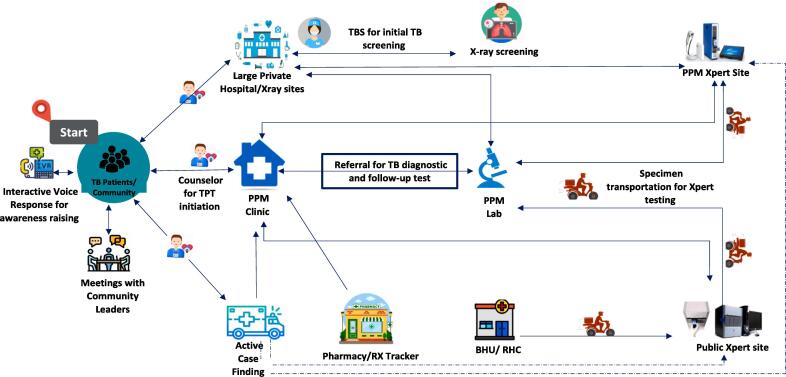
Figurative representation of various private sector engagement models being implemented by MC. Source: Mercy Corps. Overview of the private sector’s contribution in Ending TB. Meeting – Joint Program Review Mission. Unpublished; 2022.

Given that over 85 % of the population in Pakistan prefers seeking TB care from the private healthcare sector [7], engaging the private sector is crucial for improving TB care services and outcome.

Therefore, to strengthen the private sector's role in TB care, Pakistan adopted the PPM strategy in 2005 with support from the Global Fund. Over the past decade, the NTP has established four PPM models: GPs, not-for-profit health facilities, private hospitals and parastatal hospitals. Since its inception in 2002, Mercy Corps (MC), a not-for-profit international NGO has been a keen contributor to Pakistan’s TB control program, and in 2007, MC became the Principal Recipient of the Global Fund grant aimed at increasing private sector engagement in TB care [8]. Working closely with the NTP and six NGO Sub-Recipients, MC has been implementing all four models of the national PPM strategy, leading to a notable increase in TB case notifications from 33 % (in 2018) to 41 % (in 2021) [8]. Mercy Corps plays a critical role in PPM implementation in Pakistan, responsible for more than 90 % of the private sector engagement interventions. Drawing on over a decade of implementation experiences and its significant impacts on TB service delivery, this paper aims to share the various PPM models implemented by MC and their preliminary impacts on strengthening TB care services in Pakistan.

## 2 Methods

Data for this case study was sourced from MC's internal repository dating from July 2021 to December 2023. MC uses standardized data recording and reporting tools as prescribed by the National TB Control Program. Physical copies of these recording tools are housed at the respective private provider facilities and are maintained by field staff, with the support of paramedics at these facilities. All local implementing partners share a monthly progress report and supporting documents with MC teams. This data is reviewed and validated by a dedicated M&E and data management team at MC. Quarterly, a data review meeting occurs at the district level, attended by the partnering GPs and laboratory technicians, where the data is reviewed and validated by the Provincial TB Control Programs (PTPs) and Sub-Recipients (SRs). Subsequently, this data is aggregated and entered in the District Health Information Software 2 or DHIS 2 (the national NTP data reporting tool), which is further consolidated at provincial and national levels.

## 3 Mercy Corps’ PPM models

Since 2018, MC and its partners have been implementing various PPM models for assisting people with TB in early diagnosis, treatment adherence. Overall, the entire TB care management process is facilitated through a close partnership and coordination with the public sector, which offers support in supplying drugs, reagents, technical assistance and supervision.

### 3.1 Engagement of general practitioners

Under this model, MC collaborates with individual GP practices and specialists to offer TB care services at the primary care level. More than 13,000 Private healthcare providers in 120 districts across the country have been systematically engaged by MC. A systematic mapping and several dialogue sessions with individual private provider is followed by the signing of a memorandum of understanding (MoU) and training on standard national TB case management guidelines to provide free TB care services [9]. Once GPs agree to participate, MC provides them with training, data recording and reporting tools, complimentary anti-TB drugs, along with linkage to the nearest private laboratories for sputum smear microscopy and/or Xpert testing, where available. Engaged GP practices and laboratories are also incentivized (approx. 2 USD to GP for each registered case and approx. 1USD per slide for the lab) to recognize and encourage their substantial role in TB care and prevention efforts.

**Fig. 2:**
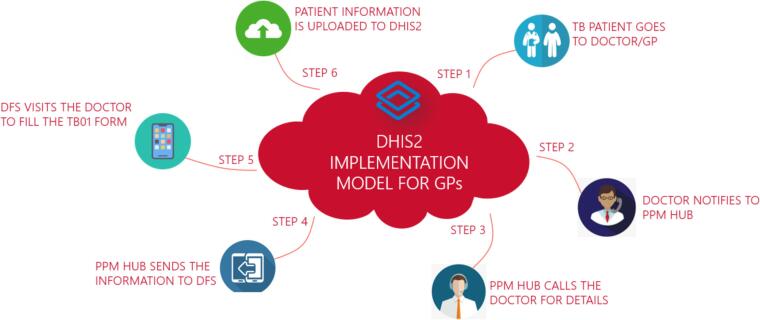
The implementation model for involving GPs using the DHIS2 platform. Source: Shah SK. 27th Annual Conference of The Union: Pakistan’s experience with building an enhanced digital TB, surveillance system [Internet]. The Union-North America Region Conference; 2023 Nov; Vancouver. Available from: https://bclung.ca/sites/default/files/2.%201025_Shah.pdf.

Since 2023, MC has also been piloting use of a call center (PPM Hub) to support GP registration, real-time patient registration, follow-up, treatment compliance, contact screening and counseling [10]. The PPM Hub is currently being piloted on a small scale with less than 3000 patients (>2% of MC total) registered via the Hub in 2023 (Fig. 2). Overall, the GP model contributed 71 % (267,753) of the total TB notifications reported by various private provider models being implemented by MC (July 2021 –December 2023) [11].

### 3.2 Engagement of private laboratories

MC has partnered with over 700 private laboratories 120 districts, based on a comprehensive mapping process, followed by dialogue sessions and signing of MoUs. In addition to providing a microscope under the Global Fund grant funds, every laboratory technician received a five-day training in sputum microscopy and Xpert testing. PTP provide laboratory reagents and other consumables.

Engagement of private laboratories ensures quality-assured diagnostic services under a hub-and-spoke model. The GP clinics (hubs) provide clinical services while the private laboratories (spokes) provide diagnostic services. The presumptive TB cases are referred from these clinics to the laboratories for further testing and diagnosis. All participating laboratories undergo External Quality Assurance mechanisms overseen by the PTP to ensure adherence to established quality standards.

### 3.3 Engagement of pharmacies

MC is employing two models to engage pharmacies. The first Model is operational across 111 districts, focuses on the referral of people with presumptive TB to the nearest public or private health facility. The second model operates under their Digital systems to Engage Private Providers in TB (DEPP TB) initiative [11], currently being piloted in 4 districts, uses a digital application called “Rx Tracker”. To reduce treatment delays and avoid deviation from standard TB case management protocols, this application tracks GPs who prescribe TB medications but are not part of the PPM network. The Rx tracker app thus captures prescriptions and traces the newly notified person with TB to their GP when a purchase is made for anti-tuberculosis (ATT) drugs at a registered pharmacy. This app builds upon the e-TB app which was developed in 2021 by the Dopasi Foundation to capture missed notifications.

### 3.4 Engagement of large private hospitals

Given the high patient influx at large private hospitals, MC has trained and recruited healthcare staff who verbally screen people visiting these hospitals for presumptive TB [9]. Known as TB Screeners, these trained healthcare workers visit different departments within the hospitals and interact with people visiting the hospital to identify TB presumptive cases. Once identified, the Screener refers them to a trained provider and in case a TB confirmation is made, the TB Screener ensures their registration, follow-up and TB treatment completion [12]. With this intervention, private hospitals with Screeners have been notifying up to 35 people with TB per quarter as compared to 8 TB notifications per quarter in other hospitals, without TB Screeners.

The increase in notifications in hospitals with Screeners can be attributed to several key factors. Screeners actively develop linkages with various units within the hospitals and encourage GPs and other specialists to refer presumptive TB cases. Additionally, Screeners conduct verbal screenings of patients visiting the hospital for other reasons, which significantly enhances case detection. This proactive approach and enhanced coordination within the hospitals contribute to the higher number of TB cases being identified and notified. In a few of these hospitals, X-ray sites are also established with the support of Global Fund, which provide free X-ray services. However, there are a total of 12 such X-ray sites in 12 districts. Similarly, there are 28 static private sector Xpert sites in 20 high burden districts, established by MC and partners which provide free-of-cost diagnostic services for TB.

### 3.5 Mobile outreach camps

Utilizing 40 mobile vans equipped with digital X-ray machines and Xpert testing, outreach teams conducted active case finding in underserved communities, with limited or no access to healthcare services. [9]. High-prevalence areas are identified using two approaches: i) public and private sector data, including TB registers and ii) MATCH AI application [13]. The MATCH AI application uses spatial, programmatic, and contextual data like poverty index and socioeconomic status, to predict the location of missed people with TB at the district and sub-district level [9], [14]. The sputum samples of individuals with presumptive TB are transported directly to either Xpert or microscopy sites from the mobile camps. Once diagnosed, the field staff takes the onus to follow up and register them at a nearby public or private health facility, aiding in timely treatment initiation and adherence. Approximately 30,000 TB cases were notified through this intervention between 2021 and 2023.

### 3.6 Specimen transportation model

**Fig. 3:**
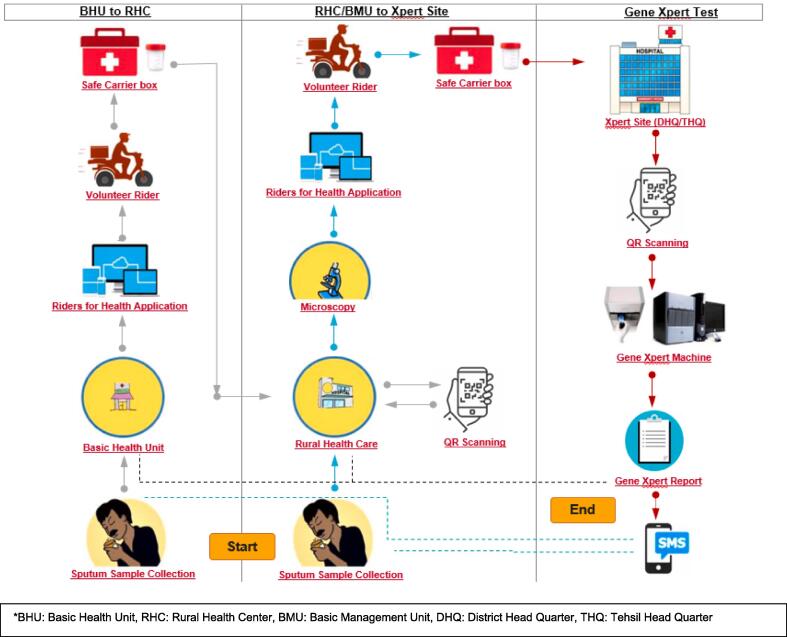
Flow of Rider Engagement through “Riders for Health” App. Source: Mercy Corps. Implementation of “Specimen Transportation Intervention (Riders for Health)” for Qualitative Diagnosis of TB - Operational Guidelines. Unpublished; 2021.

A digitally driven and robust Specimen Transportation system involving community riders has been established to strengthen the sample transportation chain (Fig. 3). With 643 registered riders, this model safeguards timely transportation of samples, contributing to diagnostic accuracy and Rifampicin Resistance (RR) case detection [14]. It pivots on an Android-based mobile application called “Riders for Health”, which moderates a ride management system (RMS) [14]. The online system provides real-time information on the number of samples transported and tested [14]. Transporting around 1,057 samples per day (Quarter 4, 2023), from both public and private healthcare facilities, this model involving riders have contributed to testing 206,147 samples at various Xpert sites (July 2021 – December 2023) [14]. Test results are shared with the provider through an exclusive interface on the Riders for Health app, while clients are notified through text about results being ready which encourages them to visit their provider for further action.

The specimen transportation model has resulted in the correct diagnosis of 7.5 % of samples on Xpert which were previously assumed negative on microscopy testing; of which 3.1 % were detected as RR-TB. Table 1 reflects the total number of samples tested on Xpert that were initially tested on microscopy and those that were tested upfront. In total, 206,147 samples were tested on Xpert, of which 17.5 % were reported as MTB detected and 2.6 % as RR-TB [15] (Table 1).

**Table 1:** Breakdown of samples tested on Xpert through ST model that were initially tested on microscopy and those that were tested upfront*.

**GX Test Detail**	**AFB Test Result**		**Up-Front GX Tests**	**Total Samples**
	Negative	Positive		
GX Tested Samples	109,236	16,438	80,473	206,147
MTB Detected Samples	8,141	14,853	13,101	36,095
	7.5 %	90.4 %	16.3 %	17.5 %
RR Detected Samples	256	370	328	954
	3.1 %	2.5 %	2.5 %	2.6 %

*Source: Mercy Corps. TB program updates – GC6. PK Global Fund TB award. Unpublished; 2024.

### 3.7 Contact screening and TB preventive treatment

Contact screening and TB Preventive Treatment (TPT) are crucial and challenging components of the PPM program. Field staff pay home visits to registered people with bacteriologically confirmed TB, interview household members to identify people with presumptive TB, and if needed, refer them to the nearest health facility [9]. After ruling out active TB, the contacts without symptoms are offered TPT. Sensitization efforts including MC’s round table meetings led by chest specialists and financial incentives for providers aim to address challenges related to prescribing TPT in Pakistan. These round table meetings are also an opportunity for GPs to share their concerns, discuss challenges and solutions [16]. Additionally, in some areas, trained counselors are appointed to counsel hesitant families and provide financial incentives to cover out-of-pocket costs for eligible household members [17]. No data was available for this intervention for the period included in paper.

**Fig. 4:**
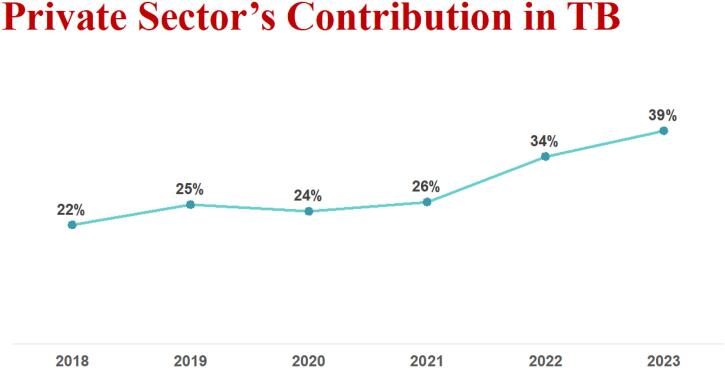
MC’s contribution in national TB case notification, over the period of six years. *Source: Mercy Corps. TB program updates – GC6. PK Global Fund TB award. Unpublished; 2024.

Overall, MC’s share in the private sector's overall contribution to TB notification in Pakistan has increased from 22 % in 2018 to 39 % in 2023 (Fig 4). Table 2 further provides the impact of each of the models discussed above in national TB case notification in 2023. Between 2020 and 2022 alone, the PPM model has successfully brought essential TB treatment closer to 267,579 people while ensuring quality care with a success rate of 93 % in 2022.

**Table 2:** Percentage Contribution of Different PPM Models in overall case notification by MC *.

**S no.**	**Model**	**Details**	**Geographical Coverage**	**Contribution in TB cases in private sector** **(2023)**
1	GP model	Solo GP practitioners/small clinics	120 districts	267,753cases (71 %)
2	Large private hospitals	Large hospitals with several specialties and more than 2 GPs including NGO-led facilities (not-for-profit small clinics/hospitals) and Parastatal Hospitals {Semi-government hospitals with defined catchment population (employees and their household Members)	76 districts	69,753cases (19 %)
4	Active case finding	Outreach camps	90 districts	32,603 cases (9 %)
5	Pharmacies engaged for referral	Drug stores	111 districts	5,199 cases (1 %)

*Source: Mercy Corps. TB program updates – GC6. PK Global Fund TB award. Unpublished; 2024.

## 4 Discussion

This paper highlights the significant impact of MC’s PPM strategies in enhancing TB care in Pakistan. By engaging over 13,000 GPs, MC’s model has made a substantial contribution to TB notifications, with GPs accounting for 79 % of notifications from private providers. The private sector’s contribution to TB notifications increased from 22 % in 2018 to 39 % in 2023. Through MC’s PPM models, over 375,308 people have been reached, achieving a 93 % treatment success rate in 2023. The deployment of TB Screeners in large hospitals has significantly enhanced TB case notifications, highlighting the effectiveness of proactive screening. Additionally, the specimen transportation model has improved access to diagnostics, enhanced diagnostic accuracy and enabled timely result reporting, leading to the detection of additional RR-TB cases.

While MC’s programs have also focused on contact screening and TB preventive treatment, data on these aspects are less comprehensive and available, and ongoing efforts are needed to address challenges in TPT and refine engagement strategies. Overall, MC’s integrated approach in leveraging private sector resources has led to substantial improvements in TB care and notification rates in Pakistan, demonstrating the effectiveness of their public–private collaboration.

Given that over 90 % of the population in Pakistan first seeks TB care from the private sector, engaging private providers is essential for improving TB care and outcomes in the country. Similar models implemented in other low- and middle-income countries have also demonstrated increased TB case notifications and improved treatment outcomes through private sector involvement.[18] For example, in Nigeria, the private sector contributed 28 % to overall case notification in 2021, and [19] in India, it accounted for around 32 % of the TB notification out of the country’s total cases [20].

Pakistan’s experience highlights the critical importance of engaging primary healthcare providers in addressing TB. However, it also emphasizes the need for an integrated approach for identifying missed TB cases and improving care outcomes. MC Pakistan began with the PPM model by collaborating with solo clinics, but later shifted to a more patient-centered approach to ensure coverage across various types of health facilities and providers. Through continuous refinement, MC developed innovative solutions, such as involving large hospitals, connecting facilities with diagnostic centers through specimen transportation to enhance accessibility, and conducting outreach camps to bring services closer to underserved communities. While each model has contributed to improved case notifications, GP clinics, given their numbers and accessibility, have had a particularly significant impact.

It is crucial to emphasize that strengthening public–private collaboration can significantly contribute to health system strengthening. The support from national and provincial TB control programs, along with other key stakeholders, was vital to the success of the PPM model in Pakistan. Although challenges remain, particularly in fully leveraging the potential of the private sector’s, this collaboration has been pivotal for the effective implementation of the PPM models. Additionally, this experience underscores the importance of tailoring interventions to the local context, as a “one size fits all” approach is ineffective. The elements of success in Pakistan offers valuable insights for similar programs in other countries. This case study also highlights the importance of involving intermediary organizations in the private sector. MC, in collaboration with six implementing partners, achieved the reported outcomes through joint efforts. Local partners on the ground played a crucial role by maintaining continuous interaction with providers, offering on-site supportive supervision, and addressing issues promptly. This hands-on approach was essential for the success of the model.

Despite these achievements, Pakistan faces challenges including the lack of enforcement and poor compliance with Mandatory TB Case Notification, despite its approval by three major provinces. This has led to deviation from standard diagnostic and treatment practices within the private sector. The low bacteriological positivity rate of 37 % (July 2021 to December 2023), in private sector is another challenge that needs attention. One of the major reasons for this low bac positivity is the insufficient use of molecular testing. This problem is further exacerbated by the limited availability of molecular testing devices in the private sector, contributing to lower RR case detection rates. To address these challenges, MC, in collaboration with key stakeholders has devised a strategy to enhance clinical diagnosis and improve bacteriological positivity rates. [21].

Another challenge is the program's sustainability, as it currently relies heavily on donor funding. The limited availability of domestic funding for TB has contributed to this dependence. The Global Fund has also highlighted the need to transition PPM initiatives to domestic funding and improve the sustainability [22].

## 5 Study limitations

One of the primary limitations of this study is the reliance on data collected exclusively by MC, which may not fully capture the broader landscape of private sector involvement in TB control initiatives across Pakistan. Additionally, while it is known that the private sector in Pakistan faces challenges such as low bac positivity rates, limited access to molecular testing, difficulties in initiating TPT, and access to newer, shorter TB regimen drugs, this study presents insufficient data to draw conclusive statements on these issues. These challenges warrant further operational research to provide deeper insights on these topics. Another limitation is the lack of assessment of the costs of TB services between the public and private sectors at the individual level. Out-of-pocket expenses remain a significant barrier to accessing TB care, despite the availability of free services.

## 6 Conclusion

MCs’ PPM models have significantly contributed to Pakistan’s progress toward achieving the End TB targets, demonstrating the effectiveness of large-scale private sector engagement within the national TB program. By increasing TB case notifications and improving treatment adherence, these models have enhanced the reach and efficiency of TB services, particularly in resource-limited settings. The case study underscores the importance of integrating private healthcare providers, showing that such collaborations can overcome traditional barriers and improve patient outcomes.

## CRediT authorship contribution statement

**Aamna Rashid:** Writing – review & editing, Writing – original draft, Resources, Project administration, Data curation, Conceptualization. **Surbhi Sheokand:** Writing – review & editing, Writing – original draft, Resources, Project administration, Investigation. **Razia Fatima:** Writing – review & editing. **Hammad Habib:** Writing – review & editing. **Adeel Tahir:** Writing – review & editing, Validation, Resources, Data curation. **Asim Saleem:** Resources, Data curation. **Poshan Thapa:** Writing – review & editing. **Petra Heitkamp:** Writing – review & editing, Supervision, Conceptualization.

## Funding

This work was supported, in whole or in part, by the Bill & Melinda Gates Foundation [INV-042531]. Under the grant conditions of the Foundation, a Creative Commons Attribution 4.0 Generic License has already been assigned to the Author Accepted Manuscript version that might arise from this submission.

## Declaration of competing interest

The authors declare that they have no known competing financial interests or personal relationships that could have appeared to influence the work reported in this paper.

## References

[1] World Health Organization. World Health Organization - Regional Office for the Eastern Mediterranean. [cited 2023 Nov 27]. WHO EMRO | Pakistan | Countries. Available from: http://www.emro.who.int/countries/pak/index.html.

[2] The World Counts. What is the Population of Pakistan [Internet]. [cited 2023 Nov 27]. Available from: https://www.theworldcounts.com/populations/countries/pakistan.

[3] World Health Organization. Global tuberculosis report 2023 [Internet]. Geneva: World Health Organization; 2023 [cited 2023 Nov 27]. Available from: https://iris.who.int/handle/10665/373828.

[4] World Health Organization. Health Service Delivery - Pakistan. Available from: https://www.emro.who.int/pak/programmes/service-delivery.html.

[5] NTP - National TB Control Program - Common Management Unit [internet]. Available from: http://www.cmu.gov.pk/ntp-national-tb-control-programme/.

[6] Ministry of National Health Services, Regulations and Coordination. National Strategic Plan For Tuberculosis Prevention, Care And Control in Pakistan 2024-2026 [Internet]. Government of Pakistan; 2024. Available from:https://cmugovpkmy.sharepoint.com/personal/support_cmu_gov_pk/_layouts/15/onedrive.aspx?id=%2Fpersonal%2Fsupport%5Fcmu%5Fgov%5Fpk%2FDocuments%2FStrategic%20Plans%202024%2D2026%2FNSP%2DFull%20Core%20plan%2DPakistan%2D12%20April%20%202023%20%2D%20V16%20%2Dfinal%20version%5Fcompressed%2Epdf&parent=%2Fpersonal%2Fsupport%5Fcmu%5Fgov%5Fpk%2FDocuments%2FStrategic%20Plans%202024%2D2026&ga=1.

[7] Engaging private health care providers in TB care and prevention: a landscape analysis, second edition. Available from: http://www.who.int/publications/i/item/9789240027039.

[8] National TB Control Program. Report of the 2022 Pakistan TB Joint Program Review Mission. Unpublished; 2022.

[9] Tahir A., Kazi G.N., Quadir A., Naureen F., Eman K.U. (2022 Jun 28). Reviewing a model of public-private mix employed for tuberculosis control in Pakistan. Pak J Public Health.

[10] Shah SK. 27th Annual Conference of The Union: Pakistan’s experience with building an enhanced digital TB surveillance system [Internet]. The Union-North America Region Conference; 2023 Nov; Vancouver. Available from: https://bclung.ca/sites/default/files/2.%201025_Shah.pdf.

[11] Mercy Corps. Overview of the private sector’s contribution in Ending TB. Meeting –Joint Program Review Mission. Unpublished; 2022.

[12] Stop TB. Pakistan: Mercy Corps implements Enhanced Case Finding approach in Punjab province to find missing people with TB [Internet]. The Strategic Initiative. [cited 2023 Dec 7]. Available from: https://stoptb-strategicinitiative.org/index.php/2018/08/07/pakistan-mercy-corps-implements-enhanced-case-finding-approach-in-punjab-province-to-find-missing-people-with-tb/.

[13] Cauwelaert CV. EPCON. 2022 [cited 2023 Dec 7]. Finding TB Cases in Pakistan: Using AI for Active Case Finding. Available from: https://www.epcon.ai/post/finding-tb- cases-in-pakistan-using-ai-for-active-case-finding.

[14] Mercy Corps. Implementation of “Specimen Transportation Intervention (Riders for Health)” for Qualitative Diagnosis of TB - Operational Guidelines. Unpublished; 2021.

[15] Mercy Corps. TB program updates – GC6. PK Global Fund TB award. Unpublished; 2024.

[16] Mercy Corps. The Operational Guidelines on Round Table District Level Advocacy Meetings. Unpublished; 2023.

[17] Mercy Corps. TB Preventive Treatment (TPT) Operational Guidelines-Programmatic Management of TB Preventive Treatment at Field Level. Unpublished; 2023.

[18] Quality of tuberculosis care in the private health sector. Available from: https://www.sciencedirect.com/science/article/pii/S2405579420300358?fr=RR-. 10.1016/j.jctube.2020.100171 PMC7332523 32642560

[19] Strategic Engagement of Private Facilities to Increase Public-Private Mix (PPM) Contribution to Nigeria Tuberculosis Case Notification. Available from: https://www.scirp.org/journal/paperinformation?paperid=119380.

[20] Exploring private sector perspectives on barriers and facilitators in availing tuberculosis care cascade services: a qualitative study from the Indian state. Available from: https://bmcprimcare.biomedcentral.com/articles/10.1186/s12875-023-02244[28]. 10.1186/s12875-023-02244-w PMC10759326 38166734

[21] Mercy Corps. “Enhancing the TB Testing on Gene Xpert and Microscopy to Reduce the Number of Clinically Diagnosed Cases and improve bac positivity”. (unpublished).

[22] Leveraging Global Fund’s investments to expand innovative public-private provider engagement in TB)”.Available from: https://www.ncbi.nlm.nih.gov/pmc/articles/PMC11249657/pdf/ijtldopen0162.pdf. 10.5588/ijtldopen.24.0162 PMC11249657 39021451

